# Solar‐Driven Hydrogen Generation Catalyzed by g‐C_3_N_4_ with Poly(platinaynes) as Efficient Electron Donor at Low Platinum Content

**DOI:** 10.1002/advs.202002465

**Published:** 2021-01-04

**Authors:** Xuan Zhou, Yurong Liu, Zhengyuan Jin, Meina Huang, Feifan Zhou, Jun Song, Junle Qu, Yu‐Jia Zeng, Peng‐Cheng Qian, Wai‐Yeung Wong

**Affiliations:** ^1^ College of Physics and Optoelectronic Engineering, Key Laboratory of Optoelectronic Devices and Systems of Ministry of Education and Guangdong Province Shenzhen University Shenzhen 518060 P. R. China; ^2^ Department of Applied Biology and Chemical Technology The Hong Kong Polytechnic University (PolyU) Hung Hom Hong Kong P. R. China; ^3^ PolyU Shenzhen Research Institute Shenzhen 518057 P. R. China; ^4^ Key Laboratory of Environmental Functional Materials Technology and Application of Wenzhou City Institute of New Materials and Industry College of Chemistry and Materials Engineering Wenzhou University Wenzhou 325035 P. R. China

**Keywords:** carbon nitrides, hydrogen generation, cocatalysts, photocatalysis, poly(platinaynes)

## Abstract

A metal‐complex‐modified graphitic carbon nitride (g‐C_3_N_4_) bulk heterostructure is presented here as a promising alternative to high‐cost noble metals as artificial photocatalysts. Theoretical and experimental studies of the spectral and physicochemical properties of three structurally similar molecules **Fo–D**, **Pt–D**, and **Pt–P** confirm that the Pt(II) acetylide group effectively expands the electron delocalization and adjusts the molecular orbital levels to form a relatively narrow bandgap. Using these molecules, the donor–acceptor assemblies **Fo–D**@**CN**, **Pt–D**@**CN**, and **Pt–P**@**CN** are formed with g‐C_3_N_4_. Among these assemblies, the Pt(II) acetylide‐based composite materials **Pt–D**@**CN** and **Pt–P**@**CN** with bulk heterojunction morphologies and extremely low Pt weight ratios of 0.19% and 0.24%, respectively, exhibit the fastest charge transfer and best light‐harvesting efficiencies. Among the tested assemblies, 10 mg **Pt–P**@**CN** without any Pt metal additives exhibits a significantly improved photocatalytic H_2_ generation rate of 1.38 µmol h^−1^ under simulated sunlight irradiation (AM1.5G, filter), which is sixfold higher than that of the pristine g‐C_3_N_4_.

## Graphical abstract

Poly(platinaynes) are used for the first time as an efficient donor for graphitic carbon nitride (g‐C_3_N_4_) to form a bulk heterojunction (BHJ) photocatalyst. This BHJ produces a sixfold higher hydrogen generation rate than that of pristine g‐C_3_N_4_. This indicates that poly(platinaynes) are a promising alternative to Pt metal cocatalysts for photocatalytic hydrogen generation at a much reduced Pt content.

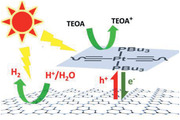

## 1 Introduction

Artificial photosynthetic fuels have attracted enormous interest due to their potential in addressing the global energy and climate crisis.^[^ ^1^ ^]^ Hydrogen (H_2_) generation from water splitting emerged as a renewable process for conversion, storage, and utilization of solar energy in an environmentally clean, economical, and efficient manner.^[^ ^2^ ^]^ A photocatalytic H_2_ generation system should be photoactive, catalytically active, and stable. Many semiconductor materials such as zinc oxide,^[^ ^3^ ^]^ titanium dioxide,^[^ ^4^ ^]^ organic dyes,^[^ ^5^ ^]^ and graphitic carbon nitride (g‐C_3_N_4_)^[^ ^6^ ^]^ have suitable energy band edges and are thus photoactive to perform light‐harvesting function, but the high overpotentials make them kinetically inert for H_2_ evolution. On the contrary, many metals and metal complexes such as platinum (Pt),^[^ ^7^ ^]^ palladium,^[^ ^8^ ^]^ cobalt,^[^ ^9^ ^]^ manganese dioxide,^[^ ^10^ ^]^ ruthenium,^[^ ^11^ ^]^ and Pt(II) complexes^[^ ^12^ ^]^ are less photoactive but catalytically active and have low overpotentials for molecular activation. A smart engineering strategy is the coassembly of a photocatalytic material and a catalytically active material to form a donor–acceptor system that drives the photocatalytic H_2_ evolution reaction. In the past decades, noble metals especially Pt metal have been widely adopted as the most efficient cocatalysts because they generally own the advantage of lower overpotential for water‐splitting than non‐noble metals despite the cost and rarity problems.^[^ ^13^ ^]^

**Figure 1:**
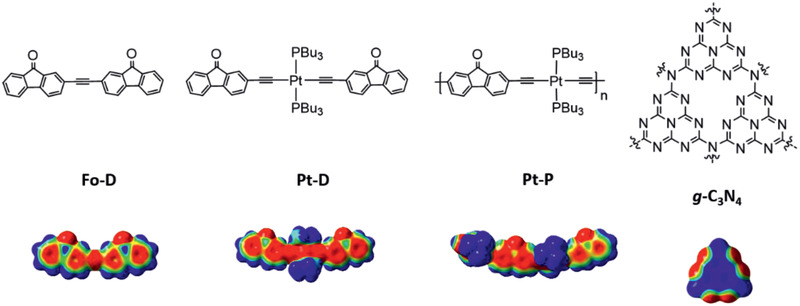
Chemical structures of **Fo–D**, **Pt–D**, **Pt–P**, and g‐C_3_N_4_ (top) and their total electron density distributions calculated using TD‐DFT (PBE1PBE/GENECP) level (bottom; blue: electron‐deficient area; red: electron‐rich area).

2D semiconductors could act as accessible *π*‐donors in composites with noble metals to form bulk heterojunction (BHJ) systems through cation–*π* interactions.^[^ ^14^ ^]^ For instance, graphene‐supported Pt and ruthenium have shown improved performance in the degradation of contaminants.^[^ ^15^ ^]^ Moreover, 2D g‐C_3_N_4_, with periodical pyridinic nitrogen atoms, is a good candidate for assembly with a metal complex.^[^ ^16^ ^]^ Abundant unsaturated nitrogen with higher electronegativity than carbon makes g‐C_3_N_4_ a Lewis base acting as a nucleophile to metal ions, which facilitates the formation of a stable BHJ system with rich photocatalytic activity. On the other hand, poly(platinaynes), generated by elaborately inserting Pt(II) bis‐acetylide into a polymeric molecular skeleton, is a highly promising electron donor for solar cell and molecular optoelectronic applications.^[^ ^17^ ^]^ Differing from Pt metal, poly(platinaynes) is characterized by a strong absorption in the visible spectral region, high charger carrier transport mobility, tunable energy band edge, and self‐assembly ability but has not been studied yet regarding their photocatalytic function. Novel composites between poly(platinaynes) and g‐C_3_N_4_ may form a new photocatalytic system with unprecedented properties for H_2_ generation. Furthermore, poly(platinaynes) only contains a relatively small amount of Pt element and can be prepared in a large area via low‐cost solution‐processed technology, resulting in cocatalysts at lower costs for practical application with respect to Pt metal on the one hand. On the other hand, the spectral property, molecular orbital energy levels, bandgap as well as relative photocatalytic function can be easily tuned by skeleton modification of poly(platinaynes), and thus it is feasible to enhance the photocatalytic activity via optimization of the molecular structure. In our work, three structurally similar and solution‐processible molecules, including the organic acetylide **Fo–D**, the Pt(II) acetylide **Pt–D**, and the poly(platinaynes) **Pt–P** (see **Figure** **1**), were synthesized and well characterized. All these molecules are *π*‐conjugated with g‐C_3_N_4_ but display different film morphologies. Among these molecules, the composite of poly(platinaynes) and g‐C_3_N_4_ with BHJ morphology and very low Pt weight ratio of 0.24% exhibits the most efficient separation of photogenerated charge carriers and an enhanced light‐harvesting capability, leading to six times improvement of the optimal photocatalytic H_2_ generation rate with respect to pristine g‐C_3_N_4_ under simulated sunlight irradiation (AM1.5G filter) without the Pt metal additive. This performance is outstanding with respect to g‐C_3_N_4_ cocatalyzed by the loading of a pure Pt metal.^[^ ^7^, ^18^ ^]^

## 2 Results and Discussion

### 2.1 Molecular and Experimental Design

With the aim to obtain a cocatalyst with good transport capability for charge carriers and broadband light‐harvesting, the “push‐pull” poly(platinaynes) molecule **Pt–P** has been designed by the introduction of an electron‐accepting fluorenone to the electron‐donating Pt(II) bis‐acetylide moiety. To reveal the effects of the Pt(II) bis‐acetylide moiety and the polymeric chain on the material's structure and properties, respectively, two other small molecules, **Fo–D** and **Pt–D**, which are structurally similar to the **Pt–P** monomer, were developed for comparison. Poly(platinaynes) is a well‐known electron donor, while g‐C_3_N_4_ is a versatile *π*‐conjugated electron acceptor.^[^ ^19^ ^]^ Molecular simulation by density functional theory (DFT) at the PBE1PBE/6‐31G(d) level by using Gaussian 09 Revision D.01^[^ ^20^ ^]^ initially revealed that the organic acetylide **Fo–D**, Pt(II) acetylide **Pt–D**, and poly(platinaynes) **Pt–P** (Figure 1, top) possess suitable molecular conformation, molecular orbital (MO) symmetry, and charge density distribution for composite formation with g‐C_3_N_4_. The total electron density distribution is shown in Figure 1 (bottom). Molecular modeling of g‐C_3_N_4_ revealed a planar conformation for heptazine with a partially positively charged center and a negatively charged periphery for electron distribution. On the contrary, molecular modeling of **Fo–D**, **Pt–D**, and **Pt–P** showed a planar conformation with a negatively charged center and a positively charged periphery for the fluorenone group. Furthermore, the metal−molecule−metal structure of Pt(II)‐acetylide *π*‐bridged fluorenone increases the electron delocalization over the whole molecule, resulting in an electron‐rich structure with a narrow bandgap (*E*_g_) to provide strong electrostatic interactions with 2D electron acceptors in addition to the *π*–*π* interactions. We assumed that these molecules form composites with g‐C_3_N_4_ based on donor–acceptor interactions via the aromatic *π* systems, which has been confirmed by calculations with Gaussian 09 Revision D.01 using DFT‐D3 at the PBE1PBE/6‐31+G(d, p) level^[^ ^21^ ^]^ (Figure S7, Supporting Information). Composite formation between **Fo–D**, **Pt–D**, or **Pt–P** and g‐C_3_N_4_ is an exothermic process with an enthalpy change (Δ*H*) of about −107.97, −96.24, and –113.42 kJ mol^−1^, respectively. Composite formation between **Pt–P** and g‐C_3_N_4_ exhibits the highest enthalpy change, implying that **Pt–P** is the strongest electron donor among the studied molecules, which enables the strongest donor–acceptor interactions with g‐C_3_N_4_. Afterwards, **Fo–D**, **Pt–D**, and **Pt–P** (1 mg each) were experimentally added to g‐C_3_N_4_ (100 mg) in chloroform solution to form BHJ photocatalysts **Fo–D**@**CN**, **Pt–D**@**CN**, **and Pt–P**@**CN**, respectively. For comparison on the photocatalytic activity, pristine g‐C_3_N_4_ was used as a benchmark and subjected to the same procedure. The synthetic details and characterization results are provided in the Supporting Information.

### 2.2 Morphological Characterization

**Figure 2:**
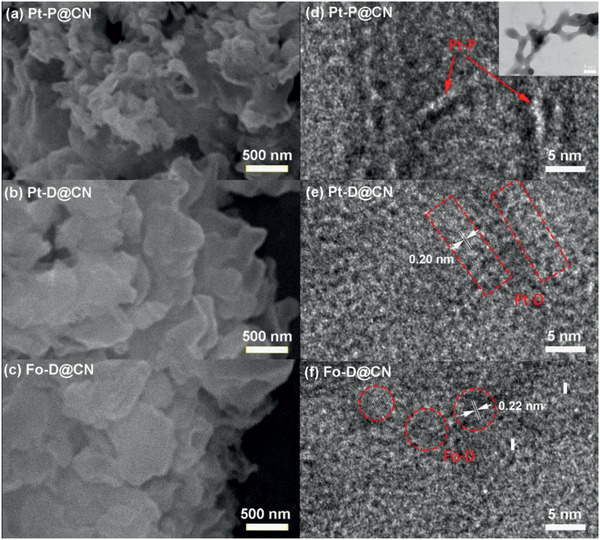
a–c) SEM and d–f) HRTEM patterns of **Fo–D**@**CN**, **Pt–D**@**CN**, and **Pt–P**@**CN**. (The inset in (d) is the HRTEM image of **Pt–P**; Scale bar: 5 nm.)

The microstructures of the BHJ photocatalysts were observed by scanning electron microscopy (SEM) and high‐resolution transmission electron microscopy (HRTEM). Interestingly, **Pt–P**@**CN** showed a petaloid‐like morphology (**Figure** **2**a), which could be ascribed to a relatively loose packing of g‐C_3_N_4_.^[^ ^22^ ^]^ In contrast, the morphologies of **Pt–D**@**CN** (Figure 2b) and **Fo–D**@**CN** (Figure 2c) are similar to that of the bulk g‐C_3_N_4_ (Figure S8a, Supporting Information), where the multilayers are relatively tightly stacked.^[^ ^23^ ^]^ This difference in morphology may originate from the self‐assembly of poly(platinaynes).^[^ ^24^ ^]^ These observations were further confirmed by HRTEM. **Pt–P** exhibited aggregates of continuous nanowires with diameters of about 2–4 nm (Figure 2d), which implies a unique bicontinuous phase structure for **Pt–P**@**CN**. From Figure 2e,f, the lattice fringe with spacing of 0.22 and 0.20 nm can be identified from the images of **Fo–D**@**CN** and **Pt–D**@**CN**, respectively. The lattice fringes should be assigned to **Fo–D** and **Pt–D**, as the reported lattice fringe spacing of g‐C_3_N_4_ is 0.33 nm, much larger than the observed spacings. The observations illustrate the successful generation of BHJ composites and both **Fo–D** and **Pt–D** were distributed in a scattered manner on g‐C_3_N_4_ without obvious aggregation. Similarly, **Pt–P**, **Pt–D**, and **Fo–D** also exhibited distinct self‐assembled structures in pristine solid state (Figure S8b–d, Supporting Information, and inset of Figure 2d), where **Pt–P** exhibited nanowires with diameters in 2–6 nm.^[^ ^25^ ^]^ Afterwards, the miscibility of these heterostructured photocatalysts was explored by energy‐dispersive X‐ray spectroscopy (EDS) elemental mapping. The obtained mapping images demonstrated uniform distributions of **Fo–D** (characterized by O), **Pt–D** (characterized by P and Pt), and **Pt–P** (characterized by P and Pt) on g‐C_3_N_4_ (Figures S10−S12, Supporting Information).

**Figure 3:**
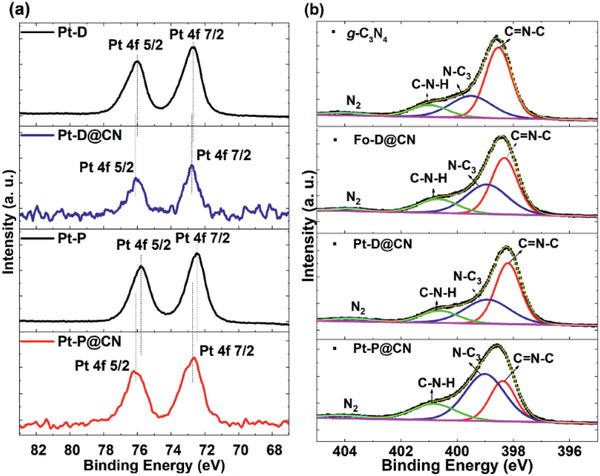
Narrow‐scan XP spectra of a) the Pt 4f core levels of **Pt–D**, **Pt–D**@**CN**, **Pt–P**, and **Pt–P**@**CN** and b) the N 1s core levels of g‐C_3_N_4_, **Fo–D**@**CN**, **Pt–D**@**CN**, and **Pt–P**@**CN**.

Furthermore, the composite surfaces were analyzed by X‐ray photoelectron spectroscopy (XPS). XPS wide‐scan spectra (Figure S14, Supporting Information) confirm that the composites contain the elements C, N, O, P, and Pt. The Pt 4f and N 1s XPS high‐resolution spectra of **Pt–D**, **Pt–D**@**CN**, **Pt–P**, and **Pt–P**@**CN** are presented in **Figure** **3**a,b. Interactions between the donor molecules and g‐C_3_N_4_ are indicated by changes in the binding energy of the Pt 4f and N 1s core levels. **Pt–D** exhibited two peaks at 72.74 and 76.04 eV, which were attributed to Pt 4f_7/2_ and Pt 4f_5/2_, respectively. After the formation of the D–A heterojunction **Pt–D**@**CN**, the Pt 4f_7/2_ and Pt 4f_5/2_ peaks were positively shifted to 72.91 and 76.28 eV, respectively. Similarly, the Pt 4f_7/2_ and Pt 4f_5/2_ peaks of **Pt–P** at 72.50 and 75.80 eV were shifted to the higher values of 72.79 and 76.10 eV, respectively, upon the formation of **Pt–P**@**CN**. The positive shift of Pt 4f XPS peaks in the composite systems unveiled the decrease in electron density, indicating the transfer of electron from Pt–D or Pt–P to g‐C_3_N_4_. On the other hand, the g‐C_3_N_4_ exhibited N 1s peak at 398.60 eV. In contrast, N 1s peak of the **Fo–D**@**CN**, **Pt–D**@**CN**, and **Pt–P**@**CN** are negatively shifted to 398.40, 398.20, and 398.50 eV, respectively, showing a slight increase in electron density of N in g‐C_3_N_4_ after forming the composites. It should be attributed to the addition of the electron donors **Fo–D**, **Pt–D**, and **Pt–P**. These findings render the successful modification of the g‐C_3_N_4_ surface with **Pt–D** and **Pt–P** based on the donor–acceptor interactions via the aromatic *π* system.^[^ ^26^ ^]^

### 2.3 Absorption Spectroscopy

**Figure 4:**
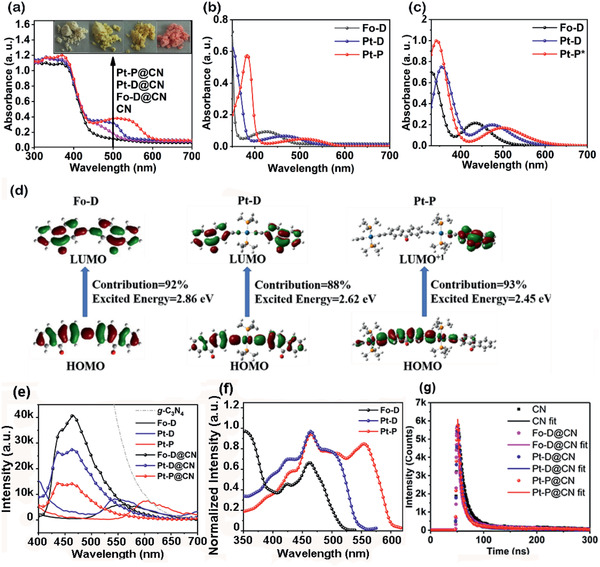
a) UV–vis diffuse reflectance spectra of solids (inset: images of g‐C_3_N_4_, **Fo–D**@**CN**, **Pt–D**@**CN**, and **Pt–P**@**CN**); b) UV–vis absorption spectra of solutions of **Fo–D**, **Pt–D**, and **Pt–P** (10 × 10^−6^ m); c) absorbance spectra of **Fo–D**, **Pt–D**, and **Pt–P** calculated by DFT at the B3LYP/GENECP level; d) main molecular orbitals involved in the electron transitions of the visible absorption peaks of **Fo–D**, **Pt–D**, and **Pt–P**; e) fluorescence spectra of BHJ photocatalysts, g‐C_3_N_4_, **Fo–D**, **Pt–D**, and **Pt–P** solids (excitation wavelength: 370 nm); f) excitation spectra of **Fo–D**, **Pt–D**, and **Pt–P** solids with scanning wavelength of 562, 544, and 602 nm, respectively. g) Lifetime decay curves of g‐C_3_N_4_, **Fo–D**@**CN**, **Pt–D**@**CN**, and **Pt–P**@**CN** solids (excitation wavelength: 370 nm).

The BHJ photocatalysts display different colors (pale yellow for g‐C_3_N_4_, yellow for **Fo–D**@**CN**, orange for **Pt–D**@**CN**, and red for **Pt–P**@**CN**) (inset of **Figure** **4**a). Their spectral properties were further studied by UV–vis diffuse reflectance spectroscopy (Figure 4a), showing good light‐harvesting capabilities with an intense absorption in the range of 300–460 nm, characteristic for g‐C_3_N_4_ and a moderate absorption in the range of 460–600 nm. The latter absorption range is characteristic for **Fo–D**, **Pt–D**, and **Pt–P**, which has been confirmed by their individual UV–vis spectra (Figure 4b, **Table** **1**). Solutions of **Fo–D**, **Pt–D**, and **Pt–P** showed two absorption peaks at 348, 350, and 382 nm as well as 426, 464, and 508 nm, respectively, with molar extinction coefficients of 7.23 × 10^4^, 6.20 × 10^4^, and 5.80 × 10^4^ m ^−1^ cm^−1^ as well as 0.93 × 10^4^, 0.66 × 10^4^, and 0.47 × 10^4^ m ^−1^ cm^−1^, respectively. Similarly, solids of **Fo–D**, **Pt–D**, and **Pt–P** (Figure S15a, Supporting Information) also exhibited two absorption peaks at 348, 350, and 382 nm as well as 451, 466, and 519 nm, respectively. The UV–vis absorptions of **Fo–D**, **Pt–D**, and **Pt–P** supported the visible light‐harvesting capabilities of the corresponding composite‐based photocatalysts. Excitation and absorption peaks of **Fo–D**@**CN, Pt–D**@**C**, and **Pt–P**@**CN** were further simulated by time‐dependent density functional theory (TD‐DFT) using Gaussian program at the PBE1PBE/GENECP level,^[^ ^27^ ^]^ and the obtained computational absorption spectra are plotted in Figure 4c. **Fo–D**, **Pt–D**, and **Pt–P** exhibited UV absorptions at 332, 354, and 344 nm and visible light absorptions at 434, 475, and 507 nm, respectively, matching with the experimental spectral data. The main contribution to the first excitation peaks, oscillator strengths, and wavelengths relative to the UV–vis absorptions are provided in Figure 4d and Figure S15b (Supporting Information), respectively, and the data are summarized in Tables S3–S5 (Supporting Information). The absorptions of **Fo–D** (332 nm, 3.73 eV) and **Pt–D** (354 nm, 3.50 eV) in the near‐UV region mainly result from the HOMO→LUMO+2 transitions with contributions of 85% for **Fo–D** and 84% for **Pt–D**. In contrast, the absorption of **Pt–P** (344 nm, 3.60 eV) is mainly originated from the HOMO−4→LUMO transitions with a contribution of ≈46%. The visible absorptions of **Fo–D** (434 nm, 2.86 eV) and **Pt–D** (475 nm, 2.62 eV) can be attributed to the HOMO→LUMO transitions with contributions of 92% and 88%, respectively. For comparison, the absorption of **Pt–P** (507 nm, 2.45 eV) is mainly ascribed to the HOMO→LUMO+1 transition with a contribution of 93%. All orbitals involved in these transitions for **Pt–D** and **Pt–P** include contributions of the Pt(II) acetylide moiety. At the same time, **Fo–D**, **Pt–D**, and **Pt–P** have increased dipole moments of 6.12, 6.25, and 6.51 a.u., respectively. These results indicate that the enhanced visible‐light absorptions of the BHJs result from the electron‐transfer characteristics of the donor molecules.

**Table 1:** Spectral and electrochemical data of **Fo–D**, **Pt–D**, and **Pt–P**

Sample	*λ*_abs_ [*ε* × 10^4^ m ^−1^ cm^−1^]			CV^a)^		
	Solution [nm]	Solid [nm]	DFT	HOMO [eV]	LUMO [eV]	*E*_g_ [eV]
**Fo–D**	348 (7.23), 426 (0.93)	348, 451	330, 435	−5.73	−3.32	2.41
**Pt–D**	350 (6.20), 464 (0.66)	350, 466	356, 474	−5.62	−3.39	2.23
**Pt–P**	382 (5.80), 508 (0.47)	382, 519	343, 499	−5.51	−3.50	2.01

^a)^ LUMO = −(4.61 + *E*_red_); HOMO = −(4.61 + *E*_ox_). Ferrocene was used as an internal standard.

The solid‐state fluorescence spectra of the pristine g‐C_3_N_4_, **Fo–D**, **Pt–D**, **Pt–P**, and the BHJ photocatalysts were obtained, as shown in Figure 4e. All the BHJ photocatalysts **Fo–D**@**CN**, **Pt–D**@**CN**, and **Pt–P@CN** exhibited emissions at around 450 nm, which should arise from g‐C_3_N_4._ It is noteworthy that the emissions, especially for **Pt–P@CN**, are noticeably weakened relative to pristine g‐C_3_N_4_. Meanwhile, the emissions from the corresponding donor molecules **Fo–D (**562 nm), **Pt–D** (544 nm), and **Pt–P** (602 nm) are negligible. These observations give an obvious evidence of fluorescence quenching due to the formation of the heterojunctions, resulting from the hole and electron transfers between the donor and acceptor units.^[^ ^28^, ^29^ ^]^ The lifetimes of the sample solids were tested by time‐resolved photoluminescence (TRPL) (Figure 4g, **Table** **2**). The afforded curves of the g‐C_3_N_4_, **Fo–D**@**CN**, **Pt–D**@**CN**, and **Pt–P@CN** were fitted with dual‐exponential model $I\;\left(t\right)=A_{1\;}\;e^{\frac{t}{t_{1}}}+A_{2\;}e^{\frac{t}{t_{2}}}$ and yielded an average lifetime (*T*) of 10.47, 7.80, 7.50, and 6.25 nm, respectively. The shortened average lifetime suggests that **Fo–D**@**CN**, **Pt–D**@**CN**, and **Pt–P@CN** have more convenient charge separation and transfer with respect to pristine g‐C_3_N_4_. Further analysis of the excitation spectra of **Fo–D**, **Pt–D**, and **Pt–P** solids (Figure 4f) revealed that **Fo–D** can be excited in both UV (≤400 nm) and visible (400−520 nm) spectral regions, while **Pt–D** and **Pt–P** are excited in broader visible spectral regions of 400–550 and 400–600 nm, respectively. The effective excitations of these three electron donors in the broad visible spectral region endow the BHJ photocatalysts with good visible light‐harvesting capability.

**Table 2:** Lifetime parameters of excitons of g‐C_3_N_4_, **Fo–D**, **Pt–D**, and **Pt–P**

Sample	*A*_1_ ^a)^	*t*_1_ ^a)^ [ns]	*A*_2_ ^a)^	*t*_2_ ^a)^ [ns]	*T* ^b)^ [ns]
CN	1.37	121.15	3655.97	9.97	10.47
**Fo–D**@**CN**	1.83	111.09	3365.15	6.89	7.80
**Pt–D**@**CN**	1.41	105.43	3365.53	6.87	7.50
**Pt–P**@**CN**	1.08	51.02	3358.49	6.13	6.25

^a)^ The fitted parameters of dual‐exponential model $I\;\left(t\right)=A_{1\;}\;e^{\frac{t}{t_{1}}}+A_{2\;}e^{\frac{t}{t_{2}}}$

^b)^ Average lifetime.

### 2.4 Electrochemical Characterization

**Figure 5:**
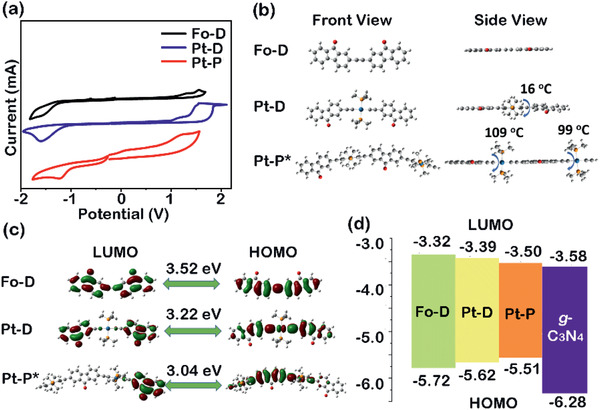
a) Cyclic voltammograms of the donor molecules (oxidative/reductive potentials of **Fo–D**, **Pt–D**, and **Pt–P** are 1.12/−1.29, 1.00/−1.22, and 0.90/−1.11 eV, respectively); b) optimal ground‐state geometries of the donor molecules, as calculated by DFT at the PBE1PBE/GENECP level using Gaussian Revision D.01 (the molecular model of polymer **Pt–P** was defined as Pt–P*); c) frontier orbital distributions and energy levels of donor molecules calculated at the same level; d) typical alignment of the frontier energy levels of donor molecules and g‐C_3_N_4_.

The electrochemical properties of **Fo–D**, **Pt–D**, and **Pt–P** were examined by cyclic voltammetry (**Figure** **5**a), revealing HOMOs/LUMOs of −5.73/−3.32, −5.62/−3.39, and −5.51/−3.50 eV, respectively, with *E*_g_ of 2.41, 2.23, and 2.01 eV. The electrochemical properties were further confirmed by DFT calculations using Gaussian 09 Revision D01 at the PBE1PBE/GENECP level. The DFT calculations at the PBE1PBE/GENECP level have been widely adopted to study the spectral and electronic properties of conjugating molecules.^[^ ^30^ ^]^ As depicted in Figure 5b, **Fo–D** has a highly planar and rigid backbone. In contrast, **Pt–D** and **Pt–P** have relatively twisted conformations around the Pt(II) acetylide unit with dihedral angles of 16° and 76°, respectively. Further analysis of the molecular orbital distributions (Figure 5c) showed that the frontier orbitals of **Pt–D** and **Pt–P** are delocalized over the Pt(II) acetylide units. Therefore, the *E*_g_ of **Fo–D**, **Pt–D**, and **Pt–P** decreased to 3.52, 3.22, and 3.04 eV, respectively. In addition, cyclic voltammetry (Figure S17, Supporting Information) reveals that the LUMO and HOMO of g‐C_3_N_4_ are located at −3.58 and −6.28 eV, respectively,^[^ ^31^ ^]^ which supports the notion that the frontier orbital energy levels of the electron donor **Fo–D**, **Pt–D**, or **Pt–P** and the electron acceptor g‐C_3_N_4_ are well aligned in the BHJs (Figure 5d).

### 2.5 Photocurrent and Impedance Tests

**Figure 6:**
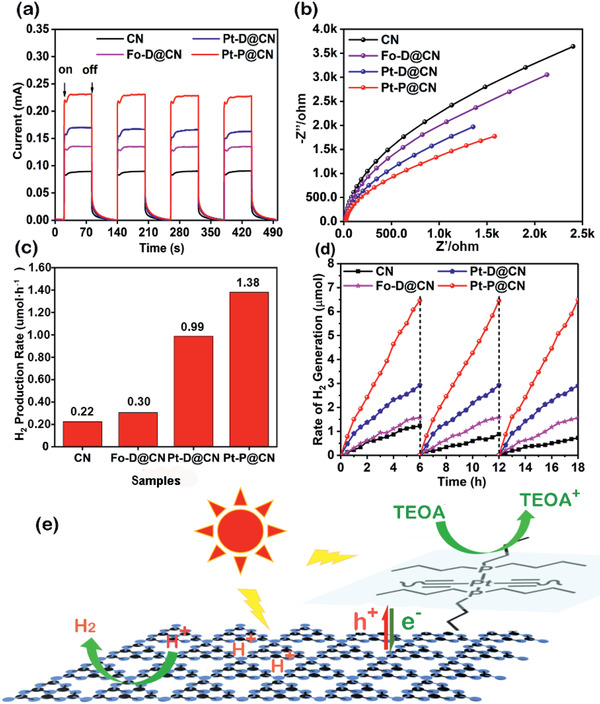
a) Photocurrent responses, b) EIS Nyquist plots, c) H_2_ generation rates, d) stability of g‐C_3_N_4_ (CN) and the BHJ photocatalysts **Fo–D**@**CN**, **Pt–D**@**CN**, and **Pt–P**@**CN**, and e) schematic illustration of the photocatalytic mechanism.

Transient photocurrent responses were recorded for several on–off cycles under intermittent irradiation using **Fo–D**@**CN**, **Pt–D**@**CN**, **Pt–P**@**CN**, or g‐C_3_N_4_ as electrode (**Figure** **6**a). The results suggest that the photogenerated electrons are effectively transferred to the back contact through the samples, resulting in a photocurrent under light irradiation.^[^ ^32^ ^]^ Compared with g‐C_3_N_4_, **Fo–D**@**CN**, **Pt–D**@**CN**, and **Pt–P**@**CN** produced higher photocurrents, supporting that the BHJ structures have better light‐harvesting capability and faster electron–hole separation rate.^[^ ^33^ ^]^ Electrochemical impedance spectroscopy (EIS) Nyquist plots obtained at a bias potential of 0.5 V (Figure 6b) showed semicircles with smaller radii for **Fo–D**@**CN**, **Pt–D**@**CN**, and **Pt–P**@**CN**, indicating lower charge transfer resistances for the BHJ structures than for g‐C_3_N_4_. At the same time, the higher photocurrent response and lower charge transfer resistance suggest a diminished electron–hole recombination, which implies that **Fo–D**@**CN**, **Pt–D**@**CN**, and **Pt–P**@**CN** would have enhanced photocatalytic activities than g‐C_3_N_4_. Furthermore, the highest photocurrent response and the lowest charge transfer resistance reveal that **Pt–P**@**CN** has the highest activity among these BHJ photocatalysts for H_2_ generation.

### 2.6 Photocatalytic Performance and Mechanism

Solar‐driven H_2_ generation tests of the BHJs **Fo–D**@**CN**, **Pt–D**@**CN**, and **PT–P**@**CN** were performed in aqueous solution using triethanolamine as a sacrificial agent under irradiation with simulated solar light (AM1.5, 100 mW cm^−2^) and without addition of Pt metal or any other cocatalyst. The apparent quantum efficiency (AQE) was detected using 3 W LEDs at the central wavelength of 420 nm and calculated based on the following equation

(1) $$\mathrm{AQE}\left(\%\right)\;=\frac{2N_{H_{2}}}{N_{p}}\;$$

where $N_{H_{2}}$ is the number of generated H_2_ molecules and *N*_p_ is the number of emitted photons. The H_2_ generation was monitored each hour by gas chromatography with thermal conductivity cell detector. Before irradiation, all the samples have been treated according to standard photocatalytic procedures in darkness, and no H_2_ generation was observed due to the photocatalytic reaction of any donor molecule **Fo–D**, **Pt–D**, or **Pt–P** without g‐C_3_N_4_ or due to any other non‐photocatalytic reaction. Figure 6c displays the photocatalytic H_2_ generation rates (HPRs) of the pristine g‐C_3_N_4_ and the BHJ photocatalysts for each 10 mg g‐C_3_N_4_ showed a relatively low HPR of 0.22 µmol h^−1^, probably due to the significant recombination of hole–electron pairs.^[^ ^6b^ ^]^ In contrast, **Fo–D**@**CN**, **Pt–D**@**CN**, and **Pt‐–‐P**@**CN** exhibited improved HPRs of 0.30, 0.99, and 1.38 µmol h^−1^, respectively. Especially, the HPR of **Pt–P**@**CN** is 6.27‐fold higher than that of the pristine g‐C_3_N_4_, which is comparable to the best performance of g‐C_3_N_4_ cocatalyzed by the generally loaded Pt metal.^[^ ^34^ ^]^ In addition, **Pt–P**@**CN** exhibited a considerable AQE of 1.43% while the pristine g‐C_3_N_4_ showed a negligible AQE at 420 nm (Figure S16, Supporting Information). Obviously, **Pt–P**@**CN** with a much less loading of Pt (0.24 wt%), holds a promise for a more economical application than pure Pt and noble metals. To test the stability, the photocatalytic experiment was continued for 18 h, showing no obvious decrease in the photocatalytic activity (Figure 6d) and thereby ascertaining the good stability of the photocatalytic system.

Based on the above discussion, a possible mechanism is proposed in Figure 6e. With tunable molecular energy band edges and visible light absorption spectra, **Fo–D**, **Pt–D**, and **Pt–P** have played a dual role as efficient electron donor in the BHJs and as light absorber complementary to g‐C_3_N_4_, which supplies more excitons and facilitates the electron transfer to g‐C_3_N_4_ when irradiated by simulated solar light. On the other hand, g‐C_3_N_4_ can also transfer its photogenerated holes to the electron donor molecule and hence suppress the significant recombination of holes and electrons. The oxidation and H_2_ evolution rates are both accelerated, and hence the photocatalytic performances of these BHJ photocatalysts are improved. Among the tested BHJ photocatalysts, **Pt–P**@**CN** with finely self‐organized behavior and the largest spectral absorption provides more valid charge carrier channels and hence shows an outstanding photocatalytic performance.^[^ ^25^ ^]^

## 3 Conclusion

In conclusion, we designed three new structurally similar molecules **Fo–D**, **Pt–D**, and **Pt–P** with organic, organometallic, and polymeric organometallic skeletons, respectively. Their spectral and physicochemical properties were comparatively studied by theoretical and experimental methods, qualitatively and quantitatively confirming that the platinaynes moiety can effectively increase the electron density, extend the electron delocalization along the molecular backbone and thus act as a strong electron donor. Furthermore, the introduction of the platinaynes unit adjusts the molecular orbital levels to form a relatively narrow *E*_g_ which thereby widens the visible light‐harvesting region. The electron donors **Fo–D**, **Pt–D**, and **Pt–P** were allowed to form composites with 2D g‐C_3_N_4_ as the electron acceptor to afford heterojunction photocatalysts. These donor–acceptor BHJ systems exhibit not only better light‐harvesting capabilities but also higher efficiencies of electron–hole separation than the pristine g‐C_3_N_4_. Especially, when used for the generation of H_2_, **Pt–P**@**CN** exhibited the highest HPR of 1.38 µmol h^−1^ for each 10 mg among all the studied BHJs, which is six times higher than that of the pristine g‐C_3_N_4_ and also surpasses those of the Pt and noble metal cocatalysts for g‐C_3_N_4_.^[^ ^34^ ^]^ This study paves the way for a new generation of highly efficient, relatively low‐cost noble metal‐based cocatalysts for solar‐driven H_2_ generation.

## Conflict of Interest

The authors declare no conflict of interest.

## Supporting information

Supporting Information
