# Supplementary material for: Solar‐Driven Hydrogen Generation Catalyzed by g‐C3N4 with Poly(platinaynes) as Efficient Electron Donor at Low Platinum Content
Source: Adv Sci (Weinh). 2021 Jan 4;8(4):2002465. doi: 10.1002/advs.202002465 (PMC7887596; doi:10.1002/advs.202002465)
Supplement: Supplementary file 1 — Supporting Information [file ADVS-8-2002465-s001.pdf]

WILEY-VCH

© 2020 Wiley-VCH GmbH

ADVANCED SCIENCE
Open Access

Supporting Information

for *Adv. Sci.*, DOI: 10.1002/advs.202002465

Solar-Driven Hydrogen Generation Catalyzed by g-C_3N_4 with Poly(platinaynes) as Efficient Electron Donor at Low Platinum Content

Xuan Zhou, Yurong Liu, Zhengyuan Jin, Meina Huang, Feifan Zhou, Jun Song, Junle Qu, Yu-Jia Zeng,* Peng-Cheng Qian,* and Wai-Yeung Wong**

Supporting Information

Solar-Driven Hydrogen Generation Catalyzed by *g*-C_3N_4 with Poly(platinaynes) as Efficient Electron Donor at Low Platinum Content

Xuan Zhou, Yurong Liu, Zhengyuan Jin, Meina Huang, Feifan Zhou, Jun Song, Junle Qu, Yu-Jia Zeng,* Peng-Cheng Qian,* and Wai-Yeung Wong**

Experimental Procedures

Materials preparation

All commercial chemicals (analytical grade) were used as received. Organic solvents were dried before use according to the standard procedures.

Theoretical calculations

The molecular geometries, the energy levels of frontal molecular orbitals, frequencies and electrostatic potentials of **Fo–D** **Pt–D** and **Pt–P** were calculated using density functional theory (DFT) at the PBE1PBE/GENECP level (Lanl2DZ basis set for Pt and 6-31g(d) basis set for the other elements) using Gaussian 09 Rev D.01. For simplicity of the calculations, methyl group was used as the side-chain substituent, and molecular fragment containing two repeating units (namely **Pt–P***) was used as the molecular model of polymer **Pt–P**. The spectral and enthalpy change calculations were performed depending on the optimized molecular geometries above with the use of time-dependent (TD)-DFT method.

Photocurrent and impedance tests

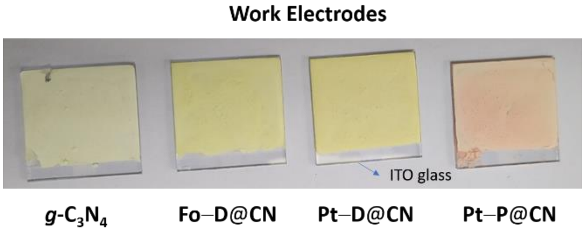

A working electrode was prepared as follows: 0.05 g of sample was ground with ethyl cellulose in ethanol to make a slurry. The slurry was coated onto an indium−tin oxide glass (ITO glass) by a doctor blade method, and then dried at 120 °C for 1 h to obtain working electrodes with a similar film thickness. The photocurrent was measured on a CHI 660E electrochemical workstation with a three-electrode system, in which the prepared electrode, a Pt (CH Instruments, Inc.) and a calomel (CH Instruments, Inc.) electrode were used as the working electrode, the counter electrode and the reference electrode, respectively. 0.5 M Na_2SO_4 solution was used as the electrolyte. A Xe lamp (CEAULIGHT, CEL-HXF300) with AM 1.5 filter (CEAULIGHT, CEL-AM 1.5) was used as the light source. The light intensity of the light source was adjusted to 50 $mW·cm^{-2}$ by adjusting the distance between the light source and the sample.

Electrochemical measurement

The cyclic voltammetry was measured on a CHI 660E electrochemical workstation with a three-electrode system, in which a glassy carbon electrode, a Pt (CH Instruments, Inc.) and a Ag/AgCl (CH Instruments, Inc.) electrode were used as the working electrode, the counter electrode and the reference electrode, respectively. 0.1 M $NBu_4F_6PO_4$ solution in DCM and ferrocene were used as the electrolyte and internal standard, respectively.

Photocatalytic experiment

10 mg of the obtained sample was dispersed in 100 ml deionized water, which contained 10 vol% of triethanolamine (TEOA). After sonication for 10 minutes, the solution was transferred to the reaction cell, and vacuumized with stirring. Xe lamp (CEAULIGHT, CEL-HXF300) with AM1.5G cut filter (CEAULIGHT, CEL-UVIRCUTAM1.5) was used as the light source (light intensity of bottom center was 100 $mW\cdot cm^{-2}$). The amount of generated hydrogen was monitored by gas chromatography (TCD, CEAULIGHT, GC-7920) every 30 minutes. Ultrapure N_2 was used as the carrier gas.

Characterization

^{1}H, ^{13}C and ^{31}P nuclear magnetic resonance spectra (NMR) were measured on an Agilent DD2-600 using $CDCl_3$ as solvent. The average molar mass and distribution were determined by gel permeation chromatography (GPC) (Waters e2695 Separations Module, Waters, Singapore), and the polystyrene samples were used as the calibration standards. The morphology of the prepared BHJs was imaged by a scanning electron microscope (SEM; thermo scientific, APREO S; EDS mapping; Bruker, XFlash 6I10) and a transmission electron microscopy (TEM; JEOL & Oxford Instruments, JEM-F200&Aztec Energy TEM SP X-MaxN 80T). Diffuse reflectance spectra (DRS) were measured using a UV-Vis spectrophotometer (PekinElmer, LAMBDA) equipped with an integrating sphere unit. The fluorescence spectra were performed at room temperature using a LS55B spectrophotometer (PerkinElmer, USA). The fluorescence decay dynamics was investigated by using an optical microscope (Edinburgh, FLS980) combined with a TCSPC module. Surface chemical states were investigated by X-ray photoelectron spectroscopy (XPS) measurement with Thermo Scientific ESCALAB 250Xi system and adventitious C1s peak (284.6 eV) as the reference. The Brunauer-Emmett-Teller (BET) of the specific surface area and pore size distributions were measured using N_2 adsorption-desorption equilibrium by Quantachrome Autosorb-1MP gas adsorption analyzer.

Synthesis of electron donors Fo–D, Pt–D and Pt–P

As illustrated in **Scheme S1**, **Fo–D**, **Pt–D** and **Pt–P** were synthesized in two steps by the Sonogashira coupling reaction. The structures were characterized by NMR, as presented in **Figure S1–S5**. The molecules **Pt–D** and **Pt–P** show excellent solubility in common organic solvents due to the herringbone side chains, while the solubility of **Fo–D** is relatively low due to its rigid skeleton. Gel permeation chromatography (**Figure S6**, **Table S1**) showed an average molecular weight of 37,776 Da and a polymer dispersity index of 1.79 for **Pt–P**.

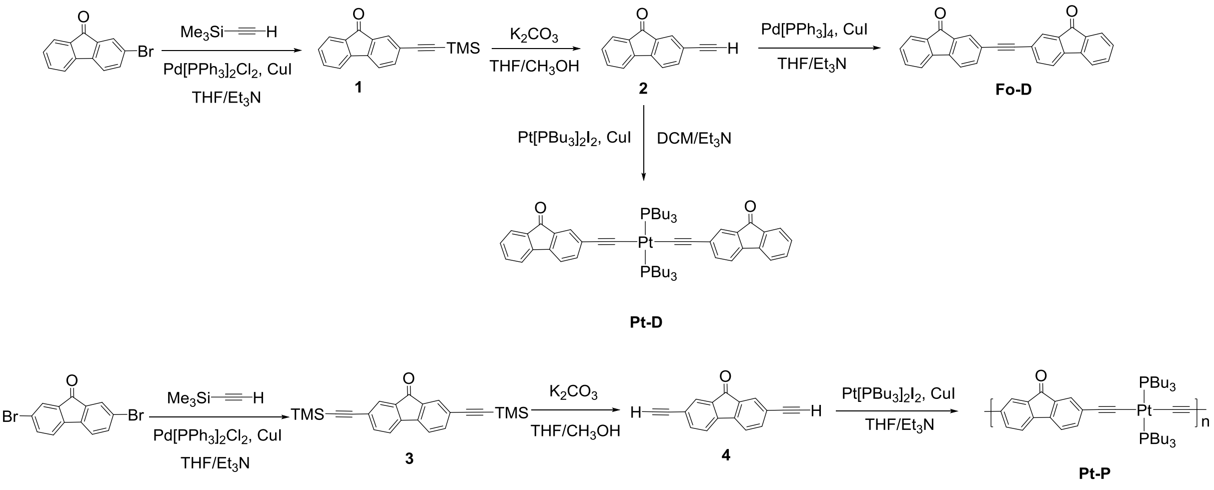

Scheme 1. Synthetic routes of molecules **Fo–D**, **Pt–D** and **Pt–P**.

Synthesis of compounds 1 and 3. 2-Bromofluoren-9-one (3.86 mmol, 1 equivalent) in 30 mL THF and 15 mL triethylamine was degassed and backfilled with nitrogen for at least three times. Bis(triphenylphosphine)palladium(II) dichloride (0.20 mmol, 2 mol% equivalent) and copper iodide (0.20 mmol, 2 mol% equivalent) were added to the flask. The mixture was bubbled with nitrogen for half an hour. Trimethylsilylacetylene (10.0 mmol mL, 10 equivalents) was injected into the flask via a syringe. The mixture was stirred for 8 hours at 50 °C and then filtered. The filtrate was concentrated under reduced pressure, and the crude product was purified by column chromatography on silica gel to give a yellow powder.

Compound **1** was obtained in 87% yield. ^{1}H NMR (400 MHz, $CDCl_3$, ppm): 7.78 (s, 1H), 7.78-7.71 (d, 1H), 7.69-7.65 (d, 1H), 7.53-7.73 (m, 3H), 7.36-7.35 (m, 1H), 0.28 (s, 9H). ^{13}C NMR (100 MHz, $CDCl_3$, ppm): 192.98, 143.92, 134.88, 134.08, 127.74, 124.04, 120.14, 104.05, 96.26, 0.12.

Compound **3** was obtained in 87% yield. ^{1}H NMR (400 MHz, $CDCl_3$, ppm): 7.75 (s, 2H), 7.60-7.58 (d, 2H), 7.47-7.45 (d, 2H), 0.28 (s, 18H). ^{13}C NMR (100 MHz, $CDCl_3$, ppm): 192.98, 143.92, 134.88, 134.08, 127.74, 124.04, 120.14, 104.05, 96.26, 0.12.

Synthesis of compounds 2 and 4. K_2CO_3 (0.99 mmol, 3 equivalents) was added to compound **2** or compound **5** (0.33 mmol, 1 equivalent) in 30 mL THF and 3 mL methanol. The mixture was stirred at room temperature for 12 hours. The resulting mixture was dissolved in 120 mL dichloromethane and washed with water, brine and finally dried over anhydrous Mg_2SO_4 to afford compound **3** or compound **6**.

Compound **2** was obtained as a yellow powder in 92% yield. ^{1}H NMR (400 MHz, $CDCl_3$, ppm): 7.78 (s, 1H), 7.71-7.69 (d, 1H), 7.65-7.63 (d, 1H), 7.55-7.37 (m, 3H), 7.36-7.35 (d, 1H) 3.19 (s, 1H). ^{13}C NMR (100 MHz, $CDCl_3$, ppm): 193.05, 143.60, 138.70, 134.92, 129.64, 124.72, 122.80, 82.76, 78.98.

Compound **4** was obtained as a yellow powder in 89% yield. ^{1}H NMR (400 MHz, $CDCl_3$, ppm): 7.79 (s, 2H), 7.60-7.64 (d, 2H), 7.53-7.51 (d, 2H), 3.21 (s, 2H). ^{13}C NMR (100 MHz, $CDCl_3$, ppm): 191.91, 143.62, 138.48, 134.33, 128.00, 123.47, 120.63, 79.23, 77.34.

Synthesis of Fo–D. 2-Bromofluoren-9-one (1.00 mmol, 1 equivalent) and compound **2** (1.00 mmol, 1 equivalent) in 30 mL THF and 15 mL triethylamine was degassed and backfilled with nitrogen for at least three times. Bis(triphenylphosphine)palladium(II) dichloride (0.20 mmol, 2 mol% equivalents) were added to the flask. The mixture was bubbled with nitrogen for half an hour. Copper iodide (0.20 mmol, 2 mol% equivalent) was quickly added to the flask. The mixture was stirred for 12 hours at 50 °C and then filtered. The filtrate was concentrated under reduced pressure, and the crude product was purified by column

chromatography on silica gel to give a yellow powder (Yield: 86%). ^{1}H NMR (400 MHz, $CDCl_3$, ppm): 7.78 (s, 2H), 7.72-7.68 (m, 4H), 7.57-7.55 (m, 6H), 7.36-7.28 (m, 2H).

Synthesis of Pt–D. Compound **2** (1.00 mmol, 1 equivalent) in 100 mL DCM and 100 mL triethylamine was degassed and backfilled with nitrogen for at least three times. Bis(tributylphosphine)platinum(II) dichloride (1.00 mmol, 1 equivalent) and copper iodide (0.20 mmol, 2 mol% equivalents) were added to the flask. The mixture was bubbled with nitrogen for half an hour. Copper iodide (0.20 mmol, 2 mol% equivalents) was added to the flask. The mixture was stirred for 12 hours at 50 °C and then filtered. The filtrate was concentrated under reduced pressure, and the crude product was purified by column chromatography on silica gel to give an orange powder. ^{1}H NMR (400 MHz, $CDCl_3$, ppm): 7.63-7.61 (d, 2H), 7.53 (s, 2H), 7.45-7.44 (m, 4H), 7.38-7.36 (m, 4H), 7.24-7.22 (m, 2H), 2.15-2.13 (m, 12H), 1.63-1.57 (m, 12H), 1.47-1.45 (m, 12H), 0.95-0.92 (m, 18H). ^{31}P NMR ($CDCl_3$, ppm): 3.63 ($^1J_{P–Pt}$ = 2,340 Hz).

Synthesis of Pt–P. Compound **4** (1.00 mmol, 1 equivalent) in 100 mL DCM and 100 mL triethylamine was degassed and backfilled with nitrogen for at least three times. Bis(tributylphosphine)platinum(II) dichloride (1.00 mmol, 1 equivalent) and copper iodide (0.20 mmol, 2 mol% equivalents) were added to the flask. The mixture was bubbled with nitrogen for half an hour. Copper iodide (0.20 mmol, 2 mol% equivalents) was added to the flask. The mixture was stirred for 12 hours at 50 °C and then filtered. The filtrate was concentrated under reduced pressure, and the crude product was purified by recrystallization in methanol to give a red powder. ^{1}H NMR (400 MHz, $CDCl_3$, ppm): 7.51 (s, 2H), 7.35-7.30 (m, 4H), 2.15-2.14 (m, 12H), 1.63-1.59 (m, 12H), 1.52-1.46 (m, 12H), 0.97-0.93 (m, 18H). ^{31}P NMR ($CDCl_3$, ppm): 3.63 ($^1J_{P–Pt}$ = 2,336 Hz).

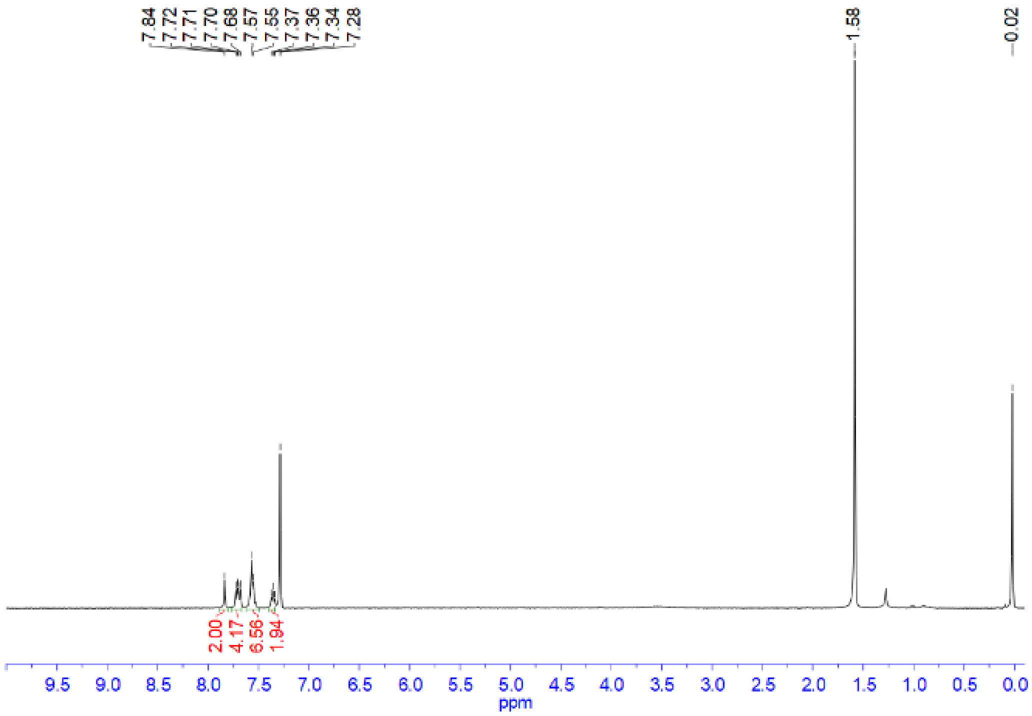

Figure S1. ^{1}H NMR of **Fo–D** in $CDCl_3$.

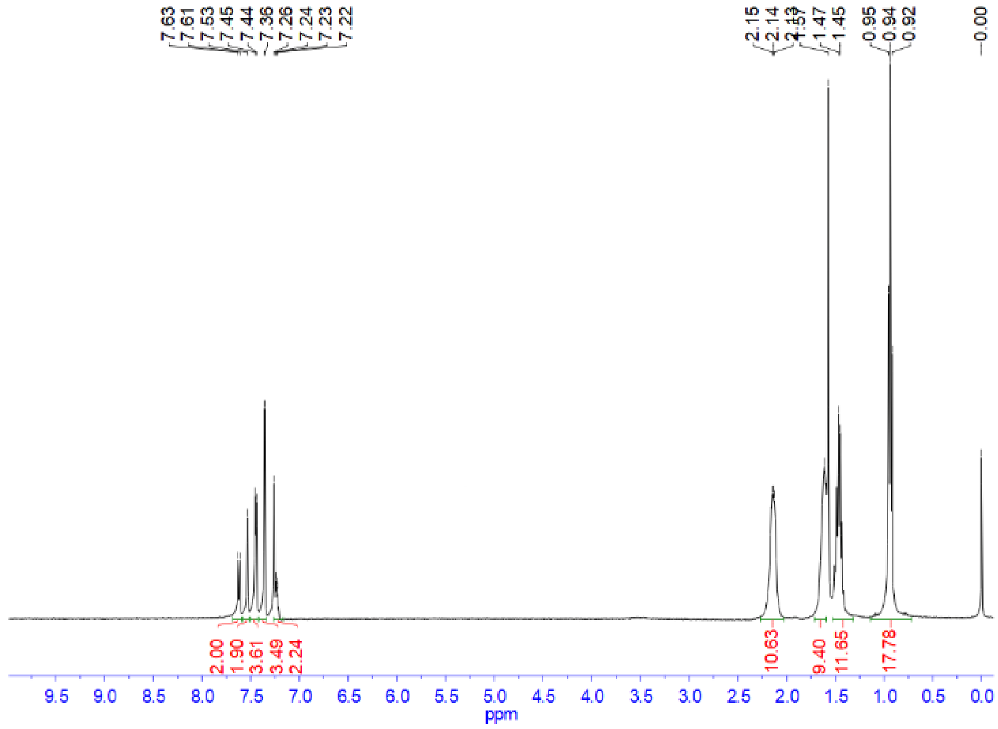

Figure S2. ^{1}H NMR of **Pt–D** in $CDCl_3$.

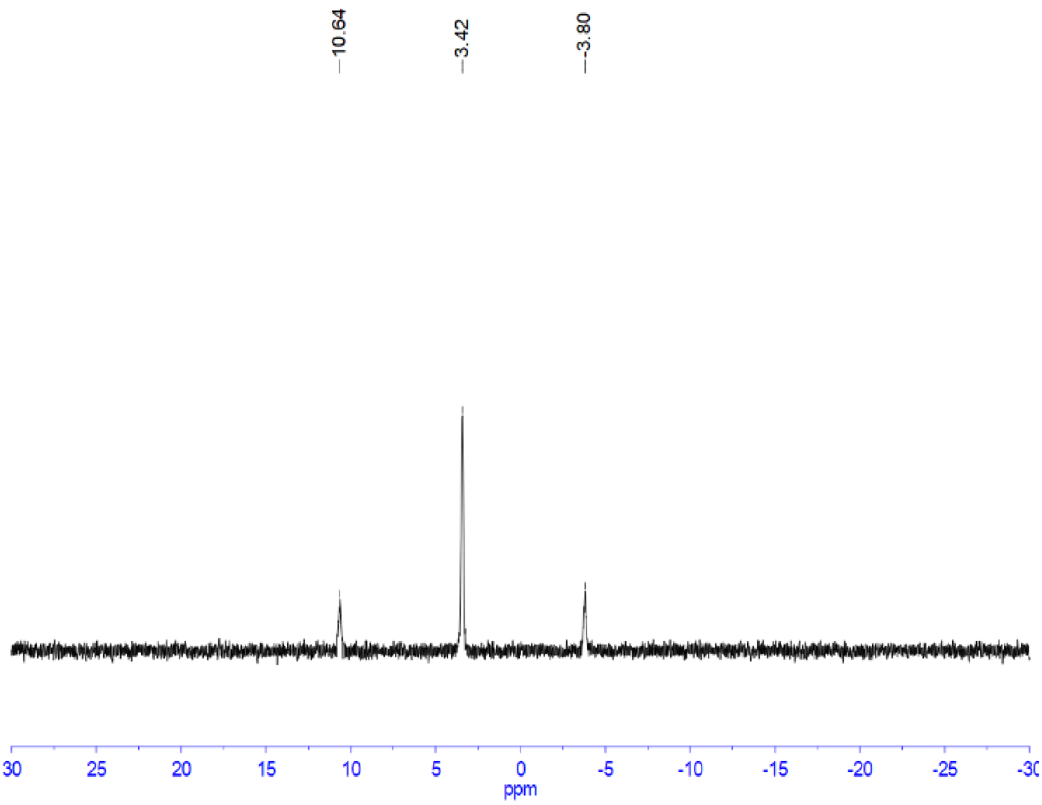

Figure S3. ^{31}P NMR of **Pt–D** in $CDCl_3$.

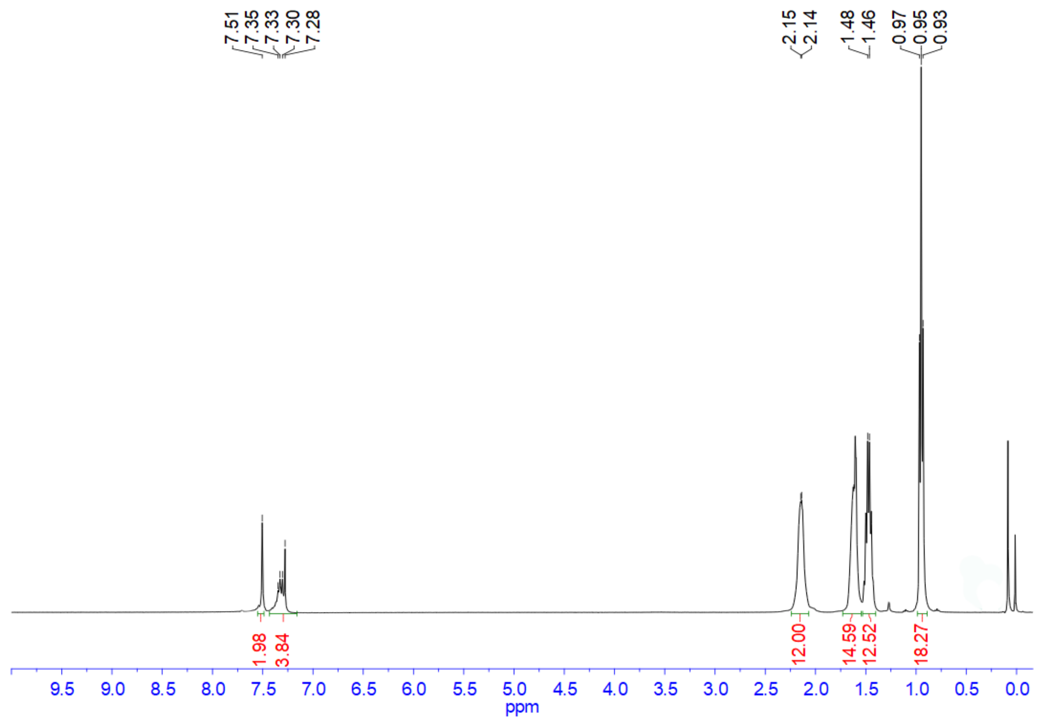

Figure S4. ^{1}H NMR of **Pt–P** in $CDCl_3$.

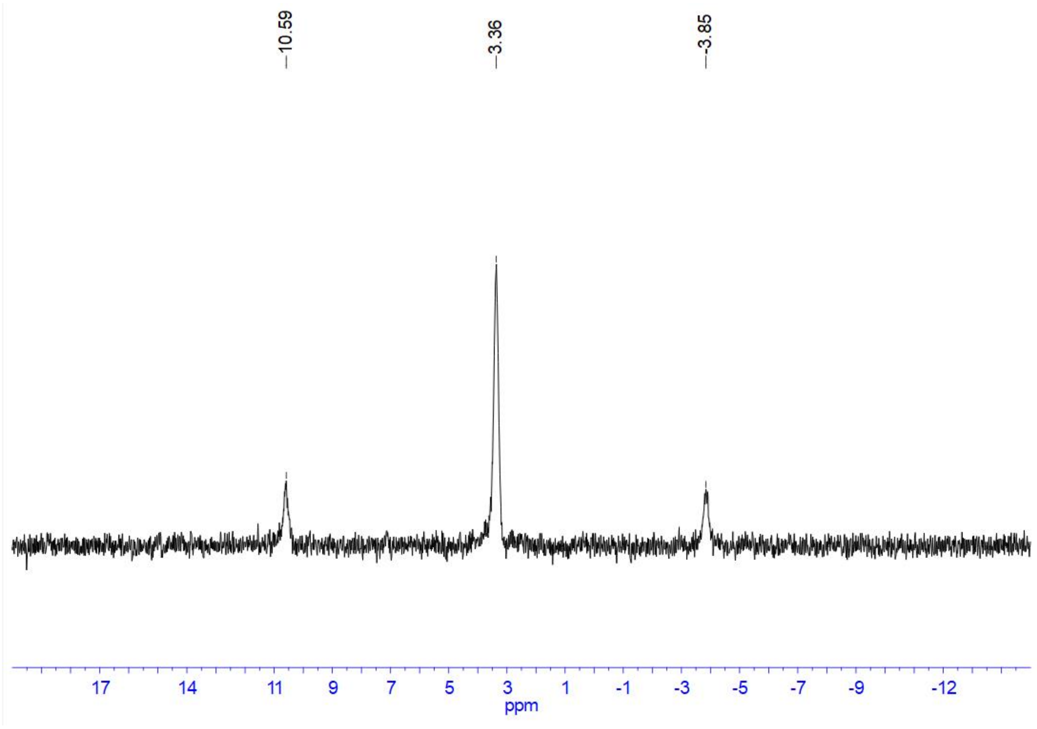

Figure S5. ^{31}P NMR of **Pt–P** in $CDCl_3$.

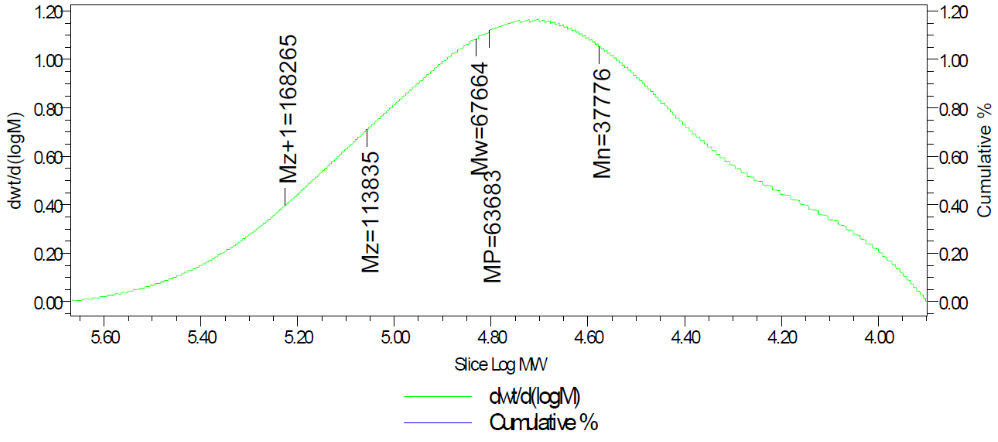

Figure S6. GPC result of **Pt–P**.

Table S1. GPC data of **Pt–P**.

Distribution	M_n[a)]	M_w[b)]	M_P[c)]	M_z[d)]	M_{z+1}[e)]	PDI[f)]
	37776	67664	63683	113836	168266	1.79

[a)] Number average molecular weight, [b)] Weight average molecular weight, [c)] Peak molecular weight, [d)] Z average molecular weight, [e)] Z + 1 average molecular weight and [f)] Polydispersity index.

Synthesis of electron acceptor *g*-C_3N_4

The electron acceptor *g*-C_3N_4 was prepared by programmed pyrolysis of urea without further purification and was obtained as a light yellow powder that is insoluble in common organic solvents.

Preparation of bulk heterojunction photocatalysts

To prepare bulk heterojunction photocatalysts, ***g*-C_3N_4** (100 mg) was sonicated in 100 mL $CHCl_3$ for 1 h to get a highly dispersed suspension. **Pt–D**, **Pt–P** or **Fo–D** (1 mg) was added to the flask, and the resultant mixture was stirred for additional 6 h until the solution was nearly clear. After solvent removal by vacuum distillation, BHJ photocatalysts were obtained as composites of *g*-C_3N_4 and **Fo–D**, **Pt–D** or **Pt–P**, denoted as **Fo–D@CN**, **Pt–D@CN** and **Pt–P@CN**, respectively. The Pt amounts in **Pt–D** and **Pt–P** were 19.39 and 23.70 wt.%, respectively, while **Fo–D** did not contain any Pt. Accordingly, the corresponding weight ratios of Pt in **Pt–D@CN** and **Pt–P@CN** were calculated as 0.19 and 0.24 wt.%, while **Fo–D@CN** did not contain any Pt. For comparison of the photocatalytic activity, pristine *g*-C_3N_4 was used as a benchmark and subjected to the same procedure.

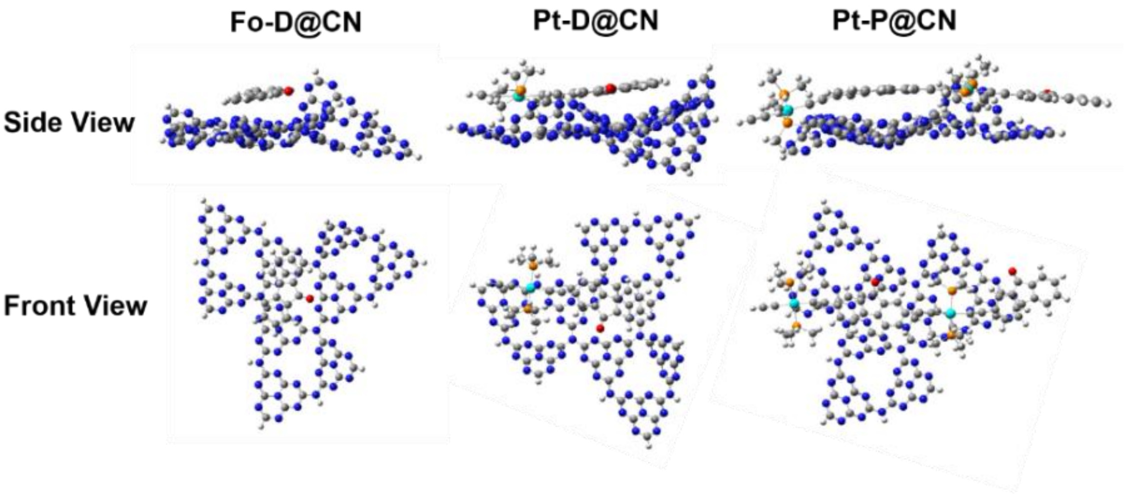

Figure S7. The DFT-D3 simulation of donor molecule and *g*-C_3N_4 calculated by Gaussian 09 D01 at the PBE1PBE/6-31+G(d, p) level.

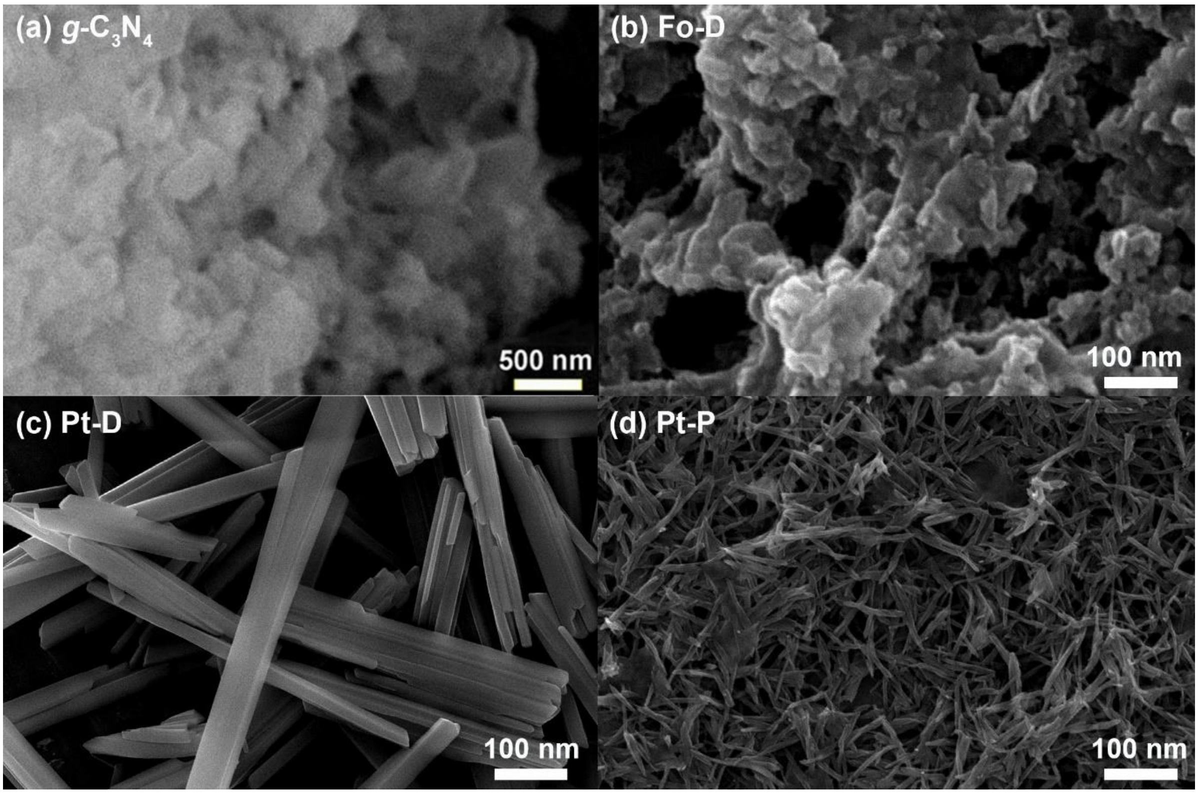

Figure S8. SEM images of ***g*-C_3N_4, Fo–D**, **Pt–D** and **Pt–P.**

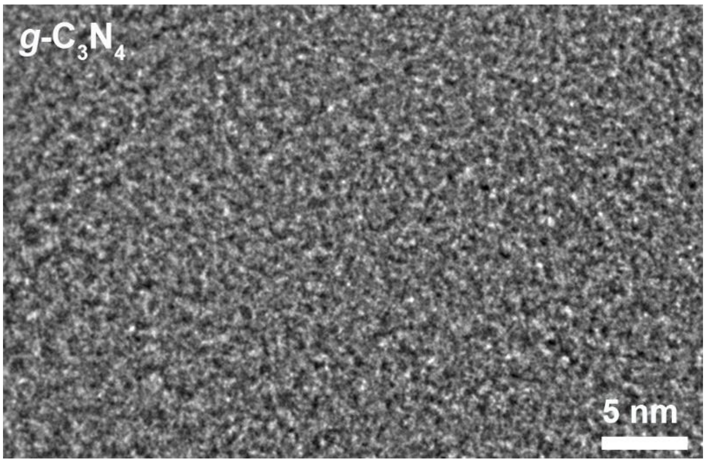

Figure S9. HR-TEM image of ***g*-C_3N_4**.

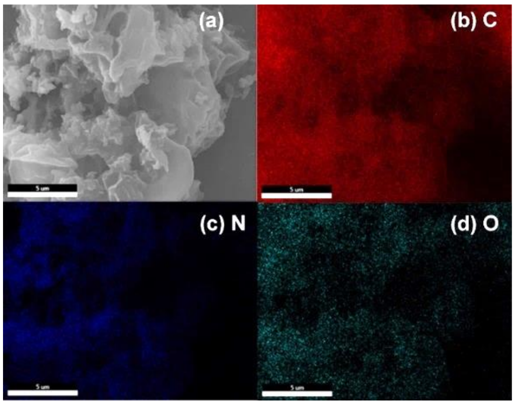

Figure S10. EDX mapping of **Fo–D@CN** on a conducting resin substrate.

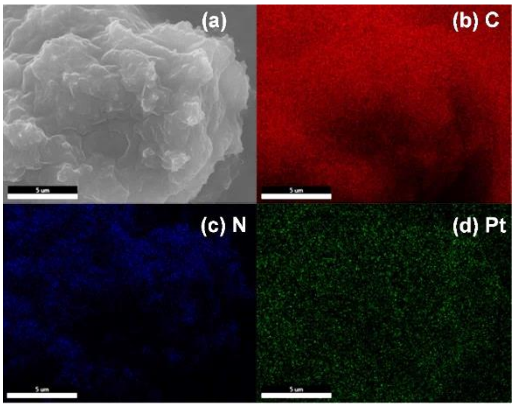

Figure S11. EDX mapping of **Pt–D@CN** on a conducting resin substrate.

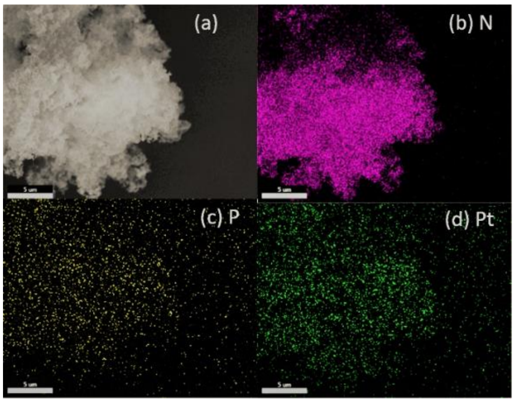

Figure S12. EDX mapping of **Pt–P@CN** on a copper foil substrate.

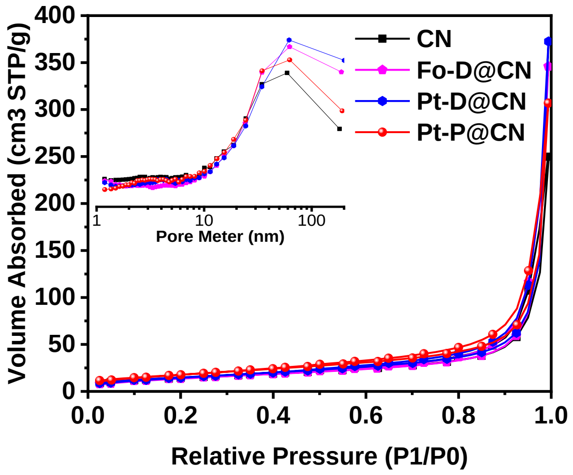

Figure S13. Nitrogen adsorption–desorption isotherms and the corresponding pore-size distribution curves (inset) of **CN**, **Fo–D@CN, Pt–D@CN** and **Pt–P@CN**.

Table S2. The BET surface area, pore volume and average pore size of **CN**, **Fo–D@CN, Pt–D@CN and Pt–P@CN**

**Samples**	**S_{BET}/ m^2·g^{-1}**	**PV[a]/ cc·g^{-1}**	**APS[b]/ nm**
**CN**	51.93	0.55	3.94
**Fo–D@CN**	52.18	0.39	1.18
**Pt–D@CN**	55.09	0.39	1.18
**Pt–P@CN**	66.25	0.48	3.19

[a] Pore volume. [b] Average pore size.

The BET test has been performed. **Figure S13** shows the nitrogen adsorption-desorption isotherms and the corresponding pore size distribution curves of the samples *g*-C_3N_4, **Fo-D@CN**, **Pt-D@CN** and **Pt-P@CN**. All the samples show isotherms of type IV according to the Brunauer–Deming–Deming–Teller (BDDT) classification and the hysteresis loops of type H3 outspread at a relative pressure range of 0.8–1, indicating the presence of mesopores (2–50 nm). Moreover, the observed hysteresis loops approach P/P0 = 1, indicating the presence of macropores (> 50 nm). The pore size distributions (inset in **Figure S13**) further demonstrate a wide distribution range from 2 to 150 nm for *g*-C_3N_4. As for the SEM images shown in **Figure S8a**, the *g*-C_3N_4 overlap each other to form a multilayer structure, resulting in numerous meso-, microporous and macropores, which are beneficial for the transportation of reactants and products. **Table S2** lists the BET surface areas, pore volumes and average pore sizes of the samples as-prepared. Both pore volumes and average pore sizes of *g*-C_3N_4 decrease with the addition of electron donor **Fo-D**, **Pt-D** and **Pt-P**. This may be due to the fact that the accumulated electron donor content can continually deposit on the surface of *g*-C_3N_4 or partly embedded in the pores, thus reducing the pore volume and average pore size. In contrast, a slight increase in the BET surface areas may be benefited from the relative weak aggregation of **Fo-D@CN**, **Pt-D@CN** and **Pt-P@CN** with respect to the pristine *g*-C_3N_4.

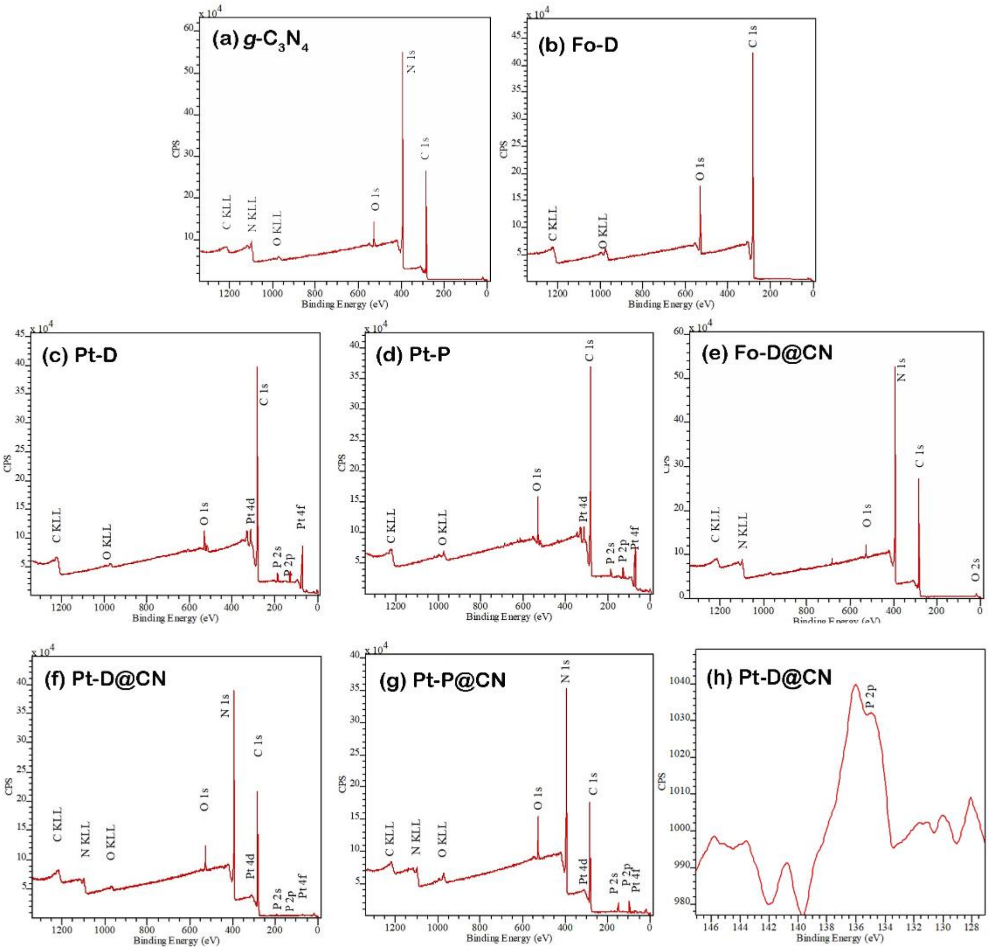

Figure S14. (a-g) The wide-scan XPS of **CN**, **Fo–D**, **Pt–D**, **Pt–P** and **BHJ** photocatalysts; (h) The magnification of the narrow-scan XPS for P 2p of **Pt–D@CN** sample.

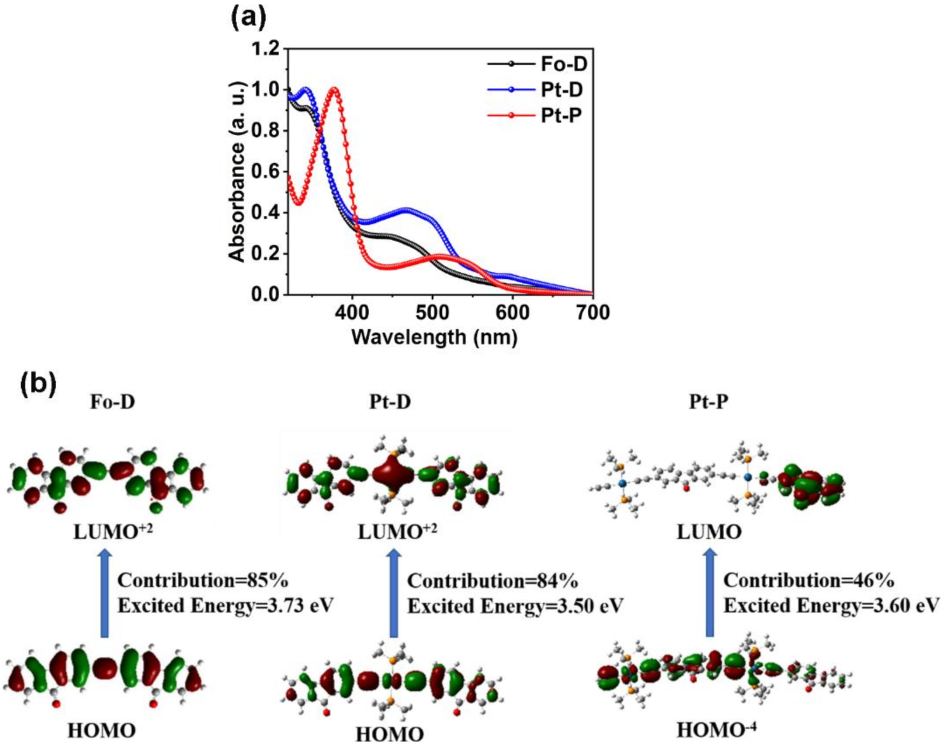

Figure S15. The main molecular orbitals involved in the excitations for UV absorption peak.

Tables S3. Selected parameters for the vertical excitation (UV-Vis absorption) of the compounds. Electronic excitation energies (eV) and oscillator strengths (f), configurations of the low-lying excited states of **Fo–D**. Calculated by TDDFT//PBE1PBE/6-31G(d), based on the optimized ground state geometries (No specific solvent was defined in all the calculations).

	Electronic transition[a)]	Excitation energy	F[b)]	Composition[c)]	CI[d)]
Absorption	$S_0 \rightarrow S_1$	2.86 eV (434 nm)	0.3726	H→L	0.67731
				H-1→L+1	0.17355
	$S_0 \rightarrow S_5$	3.73 eV (332 nm)	1.1570	H-4→L	0.10845
				H-1→L+1	0.14754
				H→L	-0.12157
				H→L+2	0.65230

a) Only selected excited states were considered. The numbers in parentheses are the excitation energy in wavelength. b) Oscillator strength. c) H stands for HOMO and L stands for LUMO. Only the main configurations are presented. d) Coefficient of the wavefunction for each excitation. The CI coefficients are in absolute values.

Tables S4. Selected parameters for the vertical excitation (UV-Vis absorption) of the compounds. Electronic excitation energies (eV) and oscillator strengths (f), configurations of the low-lying excited states of **Pt–D**. Calculated by TDDFT//PBE1PBE/GENECP, based on the optimized ground state geometries (No specific solvent was defined in all the calculations).

	Electronic transition[a)]	Excitation energy	F[b)]	Composition[c)]	CI[d)]
Absorption	$S_0 \rightarrow S_1$	2.61 eV (475 nm)	0.3394	H→L	0.66233
				H-1→L+1	-0.22361
	$S_0 \rightarrow S_7$	3.50 eV (354 nm)	1.1658	H-1→L+1	0.18496
				H-1→L+3	-0.10317
				H→L+2	0.64895

a) Only selected excited states were considered. The numbers in parentheses are the excitation energy in wavelength. b) Oscillator strength. c) H stands for HOMO and L stands for LUMO. Only the main configurations are presented. d) Coefficient of the wavefunction for each excitation. The CI coefficients are in absolute values.

Tables S5. Selected parameters for the vertical excitation (UV-Vis absorption) of the compounds. Electronic excitation energies (eV) and oscillator strengths (f), configurations of the low-lying excited states of **Pt–P**. Calculated by TDDFT//PBE1PBE/GENECP, based on the optimized ground state geometries (No specific solvent was defined in all the calculations).

	Electronic transition[a)]	Excitation energy	F[b)]	Composition[c)]	CI[d)]
Absorption	$S_0 \rightarrow S_1$	2.45 eV (507 nm)	0.2249	H→L+1	0.68166
				H→L	0.10048
	$S_0 \rightarrow S_{12}$	3.58 eV (346 nm)	0.8394	H-4→L	0.47884
				H-1→L	-0.11621
				H→L+2	0.35082
				H→L+3	0.27873
	$S_0 \rightarrow S_{13}$	3.60 eV (344 nm)	0.7819	H-4→L	0.44768
				H-4→L+1	-0.10044
				H-1→L	-0.11074
				H→L+2	-0.36194
				H→L+3	-0.30006

a) Only selected excited states were considered. The numbers in parentheses are the excitation energy in wavelength. b) Oscillator strength. c) H stands for HOMO and L stands for LUMO. Only the main configurations are presented. d) Coefficient of the wavefunction for each excitation. The CI coefficients are in absolute values.

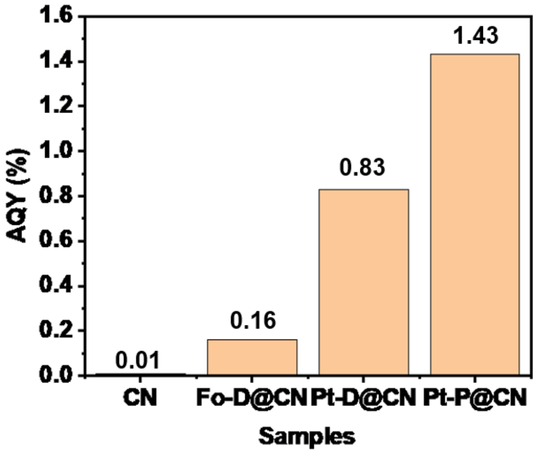

Figure S16. Comparison of the apparent quantum yields of g-C_3N_4 (CN) and the BHJ photocatalysts **Fo–D@CN**, **Pt–D@CN** and **Pt–P@CN** at 420 nm**.**

Table S6. AQE of photocatalytic H_2 generation over ***g*-C₃N₄**, **Fo–D@CN**, **Pt–D@CN** and **Pt–P@CN** under irradiation at 420 nm.

Sample	H_2 evolution (μmol)	Irradiation period (h)	AQE (%)
**g-C_3N_4**	0.05	6	0.01
**Fo–D@CN**	0.87	6	0.16
**Pt–D@CN**	4.55	6	0.83
**Pt–P@CN**	7.84	6	1.43

Reaction condition: 50 mg samples in 100 mL distilled water with 10 vol% of TEOA; Light intensity is 12.00 mW·cm^{-2}; Irradiation area is 1.20 cm^{2}.

Calculation of AQE.

The number of incident photons:

$$N_p = \frac{E\lambda}{hc} = \frac{12 \times 1.20 \times 10^{-3} \times 6 \times 3600 \times 420 \times 10^{-9}}{6.626 \times 10^{-34} \times 3 \times 10^{8}} = 6.57 \times 10^{20}$$

AQE:

$$\text{AQE} = \frac{2 \times the\ number\ of\ evolved\ H_2\ molecules}{N_p} \times 100\%$$

Then

$$AQE_{g-C_3N_4} == \frac{2 \times 6.02 \times 10^{23} \times 0.05 \times 10^{-6}}{6.57 \times 10^{20}} \times 100\% = 0.01\%$$

$$AQE_{Fo-D@CN} = \frac{2 \times 6.02 \times 10^{23} \times 0.87 \times 10^{-6}}{6.57 \times 10^{20}} \times 100\% = 0.16\%$$

$$AQE_{Pt-D@CN} = \frac{2 \times 6.02 \times 10^{23} \times 4.55 \times 10^{-6}}{6.57 \times 10^{20}} \times 100\% = 0.83\%$$

$$AQE_{Pt-P@CN} = \frac{2 \times 6.02 \times 10^{23} \times 7.84 \times 10^{-6}}{6.57 \times 10^{20}} \times 100\% = 1.43\%$$

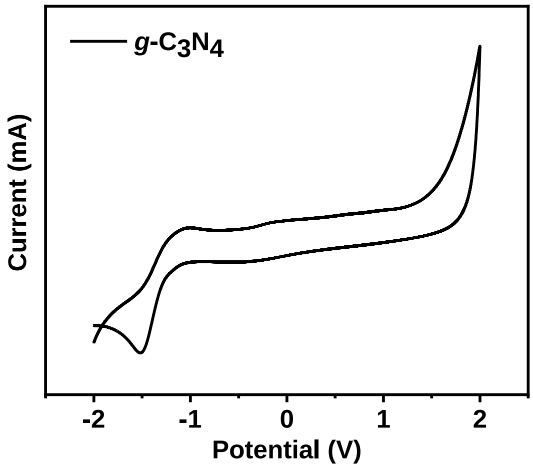

Figure S17. Cyclic voltammogram of the g-C_3N_4 (oxidative/reductive potentials are 1.03/–1.67 eV, respectively).

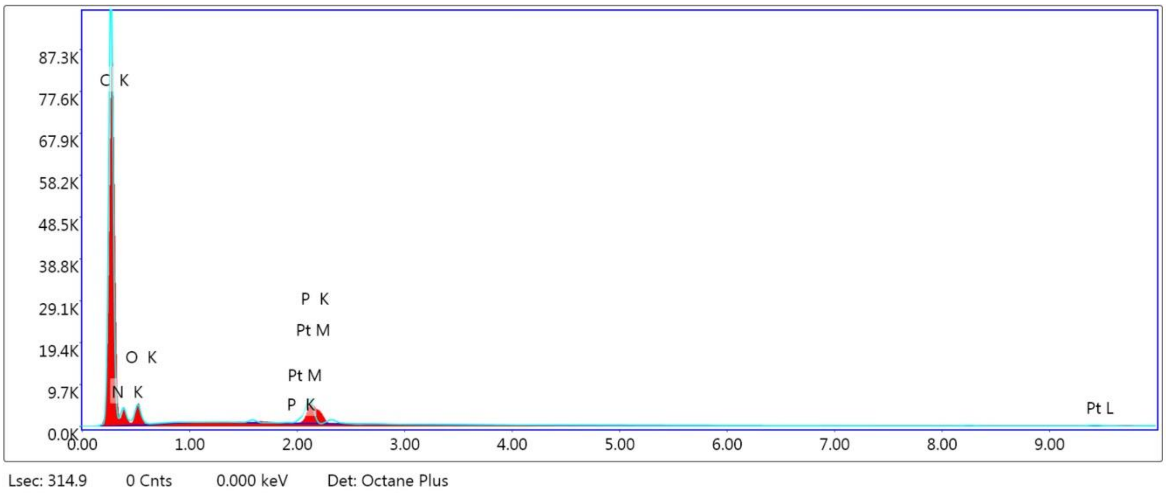

Figure S18. The energy dispersive X-ray spectroscopy (EDX) results of **Pt–D@CN**

Table S6. Energy dispersive X-ray spectroscopy data of **Pt–D@CN**

Element	*Weight (%)*	*Atomic (%)*	*Error (%)*
C K	71.02	75.60	2.95
N K	19.38	17.69	16.34
O K	8.28	6.62	15.86
P K	0.91	0.06	99.99
PtM	0.41	0.03	6.98

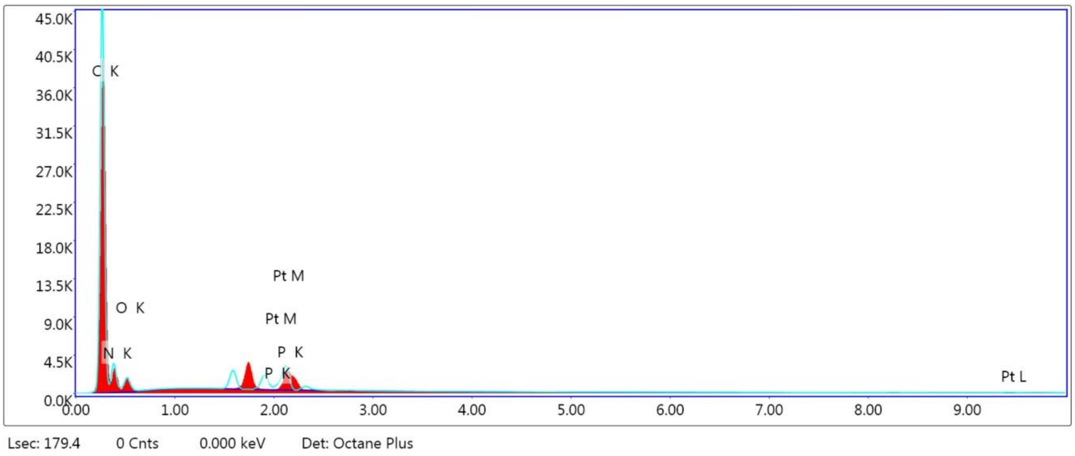

Figure S19. The energy dispersive X-ray spectroscopy (EDX) results of **Pt–P@CN**

Table S7. Energy dispersive X-ray spectroscopy data of **Pt–P@CN**

Element	*Weight (%)*	*Atomic (%)*	*Error (%)*
C K	62.85	67.30	3.05
N K	30.70	28.19	13.56
O K	5.54	4.46	19.03
P K	0.61	0.04	99.99
Pt M	0.30	0.02	6.82
